# Unusual pediatric lung infections: imaging findings

**DOI:** 10.1007/s00247-023-05818-z

**Published:** 2023-12-14

**Authors:** Domen Plut, Abbey J. Winant, Nasreen Mahomed, Kushaljit Singh Sodhi, Joanna Kasznia-Brown, Terri Williams-Weekes, Pedro Daltro, Karuna M. Das, Edward Y. Lee

**Affiliations:** 1 Faculty of Medicine, University of Ljubljana, Vrazov trg 2, 1000 Ljubljana, Slovenia; 2 Department of Pediatric Radiology, Clinical Radiology Institute, University Medical Centre Ljubljana, Zaloška cesta 2, 1000 Ljubljana, Slovenia; 3 Department of Radiology, Boston Children’s Hospital and Harvard Medical School, Boston, MA USA; 4 Department of Radiology, University of Witwatersrand, Johannesburg, South Africa; 5 Mallinckrodt Institute of Radiology, Washington University in St. Louis School of Medicine, St. Louis, MO USA; 6 Department of Radiodiagnosis, PGIMER, Chandigarh, India; 7 University of Bristol, Musgrove Park Hospital, Taunton, UK; 8 Tufts Medical Center, Boston, MA USA; 9 Department of Radiology, Clínica de Diagnóstico por Imagem, Rio de Janeiro, Brazil; 10 Department of Radiology, College of Medicine and Health Sciences, United Arab Emirates University, Al Ain, United Arab Emirates

**Keywords:** Chest, Child, Computed tomography, Infection, Pneumonia, Radiography, Ultrasound

## Abstract

Pediatric lung infections continue to be a leading cause of pediatric morbidity and mortality. Although both pediatric and general radiologists are familiar with typical lung infections and their imaging findings in children, relatively rare lung infections continue to present a diagnostic challenge. In addition, the advances in radiological imaging and emergence of several new lung infections in recent years facilitated the need for up-to-date knowledge on this topic. In this review article, we discuss the imaging findings of pediatric lung infections caused by unusual/uncommon and new pathogens. We review the epidemiological, clinical, and radiological imaging findings of viral (coronavirus disease 2019, Middle East respiratory syndrome, bird flu), bacterial (*Streptococcus anginosus*, *Francisella tularensis*, *Chlamydia psittaci*), and parasitic lung infections (echinococcosis, paragonimiasis, amoebiasis). Additional disorders whose clinical course and imaging findings may mimic lung infections in children (hypersensitivity pneumonitis, pulmonary hemorrhage, eosinophilic pneumonia) are also presented, to aid in differential diagnosis. As the clinical presentation of children with new and unusual lung infections is often non-specific, imaging evaluation plays an important role in initial detection, follow-up for disease progression, and assessment of potential complications.

**Graphical Abstract:**

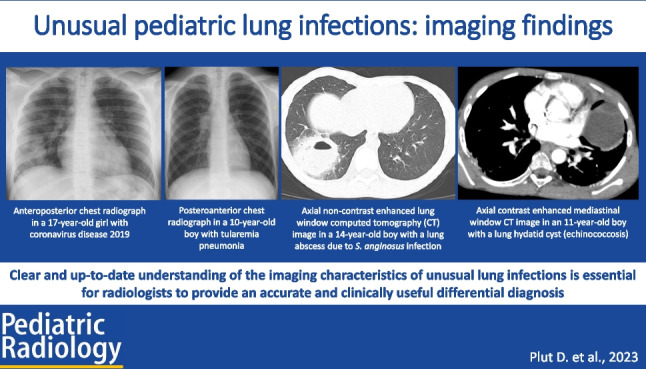

## Introduction

Pediatric lung infection continues to be a leading cause of pediatric morbidity and mortality [1]. Although both pediatric and general radiologists are familiar with typical common lung infections and their imaging findings in children, relatively rare lung infections continue to present a diagnostic challenge. Due to migration and wide-spread global tourism, once endemic infections can now be encountered anywhere in the world [2]. In addition, in recent years, there have been outbreaks of several new viral lung infections [3–5]. Radiologists anywhere may encounter these diseases in their daily practice and require up-to-date knowledge for accurate diagnosis and disease follow-up. Therefore, the goal of this review article is to discuss the imaging findings of pediatric lung infections caused by unusual/uncommon and new pathogens. Additional disorders whose clinical course and imaging findings may mimic lung infections in children are also presented to aid in differential diagnosis.

## Currently available imaging modalities and their roles in evaluation of pediatric lung infection

Guidelines vary on the role of imaging in the initial diagnosis of pediatric lung infections. The clinical practice guidelines by the Pediatric Infectious Diseases Society and the Infectious Diseases Society of America state that routine chest radiography is not necessary for the confirmation of suspected lung infection in children well enough to be treated in the outpatient setting (i.e. after evaluation in the office, clinic, or emergency department setting) [6]. Similarly, the guidelines of the British Thoracic Society for the management of lung infection in children state that chest radiography should not be considered a routine investigation in children thought to have community acquired pneumonia [7]. However, according to these guidelines, chest radiography should be obtained in all patients hospitalized for management of community acquired pneumonia and in those who have failed initial antibiotic therapy. Follow-up chest radiography should be obtained in children who fail to demonstrate clinical improvement and in those who have progressive symptoms or clinical deterioration.

In children with parapneumonic effusion suspected by physical examination or chest radiography, further evaluation with chest ultrasound or computed tomography (CT) may be needed. Chest ultrasound is considered a safer imaging modality than CT due to lack of ionizing radiation [6, 7]. Ultrasound is an excellent imaging modality for detection and quantification of pleural effusions. Chest ultrasound can precisely diagnose size, echotexture, complexity, and loculation of pleural fluid, which can be helpful for differentiating transudative effusion from exudative effusion (e.g., empyema) with sensitivity up to 90% [8, 9]. Thoracentesis with pleural fluid analysis can be confirmative for definitive histopathologic diagnosis of pleural fluid [8]. Ultrasound can also detect lung necrosis or abscess formation in the setting of complicated pneumonia [9]. CT is typically reserved for advanced evaluation of complicated lung infections if ultrasound is technically limited, discrepant from clinical findings, or if surgical intervention is planned [9, 10]. In recent years, contrast-enhanced ultrasound (CEUS) has emerged as a valuable complementary ultrasound technique that can offer additional information to gray-scale and Doppler chest ultrasound. There is limited but growing experience in the use of CEUS for the evaluation of complicated pneumonia [11–13]. Due to its lack of ionizing radiation and its ability to be performed at-the-bedside, CEUS is a promising modality for evaluation of pediatric chest infections in the future.

Chest radiographs demonstrating absent, minimal, or atypical findings in a pediatric patient with symptoms of respiratory infection can suggest the possibility of unusual lung infection. Once unusual lung infection is suspected either clinically or radiographically, a thorough review of the patient’s clinical manifestations, exposure risk factors, and laboratory workup test results is necessary to narrow the diagnostic possibilities and determine the appropriate next steps in management. In these cases, chest CT can be a valuable diagnostic tool. CT provides greater anatomic detail and is more sensitive for detection of infection than chest radiography. Therefore, the indications for chest CT in these pediatric patients may be broader than just the depiction of pulmonary complications [14]. As microbiologic isolation of unusual microorganisms can be challenging and clinical symptoms are often nonspecific, a multifactorial approach that combines environmental exposure information, risk factors, and CT findings can enhance diagnostic accuracy, enable earlier initiation of proper treatment, and prevent potential complications.

## Spectrum of unusual lung infections in children

Although there is a myriad of unusual lung infections in children, the following sections discuss selected disorders that are currently relevant in daily clinical practice. Table 1 summarizes the key radiographic and CT features of all the described diseases.

**Table 1:** Typical radiographic and computed tomography findings of unusual lung diseases and their mimics

Pathogen/disease	Radiographic findings	CT findings
*Coronavirus disease 2019 (COVID-19)*	• Can often appear normal • Unilateral or bilateral peripheral and/or posterior, and lower lobe-predominant hazy opacities with or without consolidation	• Unilateral or bilateral peripheral and/or posterior, and lower lobe-predominant ground-glass opacities with or without consolidation • Halo sign • Reversed halo sign • Crazy-paving pattern
*Middle East respiratory syndrome (MERS)*	• Unifocal subtle opacities in the middle and basal regions of the lungs • Progress to multifocal and bilateral airspace opacities	• GGOs alone or in combination with consolidation in a peripheral and basilar distribution involving multiple lung segments
*Bird flu*	• Reticular opacities with areas of patchy consolidations, confluent on repeated chest radiographs • Multifocal and bilateral, with a predilection for the lower lobes • Bilateral pleural effusion and cavitations can appear	• Multifocal or diffuse GGOs with areas of consolidations • Bilateral pleural effusion and cavitations can appear • During the convalescent stage can show signs of post-infective fibrosis
*Streptococcus anginosus group*	• Consolidations • Lung abscesses • Pleural effusion	• Consolidations complicated by lung necrosis • Lung abscesses • Pleural effusion (usually complex)
*Tularemia*	• Uni/bilateral lung opacities • Hilar lymphadenopathy • Pleural effusion	• Peripheral dense lobar lung consolidation • Lymphadenopathy • Lung abscess
*Psittacosis*	• Bilateral large areas of consolidation • Pleural effusion	• Bilateral large areas of consolidation or GGOs involving multiple lobes • Pleural effusion
*Echinococcosis*	• Well-defined homogenous opacity, more commonly appearing in the lower lobes	• Cysts can have calcified walls • Can show bronchial erosion • Can be multiple and bilateral
*Paragonimiasis*	• Patchy pulmonary consolidations • Pleural effusion • Hilar lymphadenopathy	• Ill-defined pulmonary nodules • Consolidation • Cystic lesions filled with fluid or gas • Surrounded by GGOs
*Amoebiasis*	• Consolidation with pleural effusion • Abscess • Right lower lobe is most commonly involved	• Amorphous abscess with thick irregular walls and with or without an air-fluid level • Pleural effusion
*Hypersensitivity pneumonitis*	• Hazy opacities throughout both lungs • Sometimes with sparing of the apices and bases • A pattern of fine reticulation may occur	• Patchy or diffuse bilateral GGOs • Small centrilobular nodules • Mosaic attenuation • Air trapping • Chronic/fibrotic form: thickened interlobular septa, traction bronchiectasis, and subpleural honeycomb pattern • Sparing of the lung bases
*Pulmonary hemorrhage*	• Focal: patchy alveolar opacities, dense consolidation, atelectasis • Diffuse: symmetric diffuse hazy pattern, predominates in the lower lung zones with sparing of the apices and costophrenic angles	• Can depict the underlying disease • Focal: patchy GGOs, dense consolidation • Diffuse: symmetric diffuse ground-glass pattern, predominates in the lower lung zones with sparing of the apices and costophrenic angles
*Idiopathic eosinophilic pneumonia*	• Acute: diffuse interstitial and air-space opacities without peripheral predominance • Chronic: reverse batwing appearance	• Acute: diffuse asymmetric GGOs • Chronic: GGOs, pulmonary nodules, reticulation

*CT* computed tomography, *GGO* ground-glass opacity

### Viral infections

#### Coronavirus disease 2019

Coronavirus disease 2019 (COVID-19) is caused by the severe acute respiratory syndrome coronavirus 2 (SARS-CoV-2), a single-stranded RNA virus belonging to the β-coronavirus cluster [15]. The primary mode of transmission for SARS-CoV-2 is through respiratory droplets, which allows rapid spread of the disease. Once the virus is inhaled, it binds to ACE2 receptors on alveolar epithelial cells and leads to cell damage that results in alveolar destruction [16]. This eponymous “unusual (COVID-19) pneumonia” rapidly spread and resulted in a global pandemic, with over 700 million confirmed cases and almost 7 million deaths [17]. The elderly were most severely affected, while children overall demonstrated a milder clinical course with rare cases of severe pneumonia. In children, a post-viral multisystem inflammatory syndrome (MIS-C) was responsible for a more serious clinical course of the disease. This difference may relate to lowered expression of ACE2 receptors in nasal and bronchial tissue in pediatric patients as well as differences in immune system maturity [18].

**Fig. 1:**
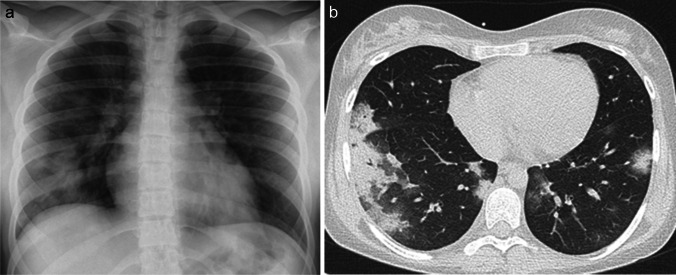
Images in a 17-year-old girl with coronavirus disease 2019 (COVID-19) who presented with fever, cough, and fatigue. **a** Anteroposterior supine chest radiograph shows bilateral peripheral opacities predominately in the basal segments of the lungs. **b** Axial non-contrast-enhanced lung window computed tomography image shows the typical signs of COVID-19 in children: subpleural and peripheral consolidations in basal and posterior lung segments surrounded by ground-glass opacities (the halo sign)

**Fig. 2:**
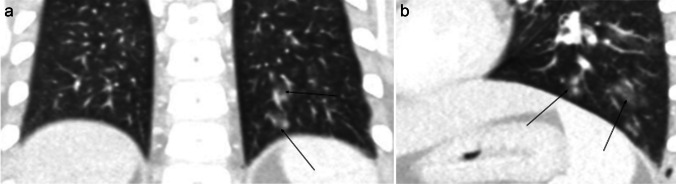
Coronal (**a**) and sagittal (**b**) contrast-enhanced lung window computed tomography images in a 7-year-old girl with coronavirus disease 2019 show small ground-glass opacities (*arrows*) in the basal and posterior lung segments – these findings were not visible on radiography

The clinical presentation and blood laboratory analysis of COVID-19 pneumonia is relatively nonspecific. Cough, fever, myalgia, and fatigue are the most common presenting clinical features. On imaging (Figs. 1 and 2), COVID-19 pneumonia in pediatric patients appears as unilateral or bilateral peripheral (subpleural) and/or posterior, and lower lobe-predominant hazy opacities with or without consolidation [19–22]. These findings can be detected on chest radiography; however, chest CT is more sensitive for detection and localization of the imaging findings of COVID-19 pneumonia, especially subtle ground-glass opacities which can only be detected on CT [23–26]. In addition, the CT halo sign is also considered a typical finding of the early phase of pediatric COVID-19 pneumonia [20]. However, the additional CT findings do not affect management in patients with mild to moderate symptoms; therefore, CT is not indicated in these patients and is only typically utilized in patients with a severe COVID-19 pneumonia [25]. Atypical imaging findings, including centrilobular nodules (including tree-in-bud nodularity), cavitation, pleural effusion, and/or lymphadenopathy, are uncommon findings in pediatric COVID-19 pneumonia. Consequently, when present, these atypical imaging features should raise strong consideration for alternative diagnoses [20].

#### Middle East respiratory syndrome

Middle East respiratory syndrome (MERS) is a viral respiratory infection caused by Middle East respiratory syndrome coronavirus (MERS-CoV), also a single-stranded RNA virus belonging to the β-coronavirus cluster [27]. First identified in Saudi Arabia in September 2012, MERS led to a pandemic that spread around 27 countries [28]. MERS-CoV primarily binds to the cell surface protein dipeptidyl peptidase 4 (DDP4), which is abundant in the human respiratory tract. However, DDP4 is also expressed by epithelial cells in the kidneys, small intestines, liver, prostate, and activated leukocytes. MERS-CoV subsequently leads to immune dysregulation, postulated to result in a delayed proinflammatory response in lung epithelial cells [15].

The clinical features of MERS range from asymptomatic or mild disease to acute upper respiratory illness, rapidly progressive respiratory failure with septic shock, multiorgan failure, and death. Although patients of all ages can be affected, the symptoms of MERS are the same across age groups, including fever, dyspnea, and cough [29]. Like COVID-19, children are less commonly affected and typically present with a mild clinical course. Similar to COVID-19, poor outcome is more common in children with concomitant medical comorbidities [30].

Chest radiography is an important imaging modality for both initial evaluation and following disease progression. The most frequently observed chest radiography findings in patients with MERS are unifocal subtle opacities in the middle and basal regions of the lungs, which may eventually progress to multifocal and bilateral airspace opacities as the disease progresses [31]. A fine interstitial reticular pattern of interstitial inflammation has been described in mild disease with progression into diffuse bilateral opacities mixed with air space consolidation in severe illness [5, 30]. On CT, the most frequently observed pattern is ground-glass opacities alone or in combination with consolidation in a peripheral and basilar distribution involving multiple lung segments in more seriously ill patients [32].

#### Bird flu

Bird flu, also known as avian influenza or avian flu, is a bird disease caused by the influenza A virus. Although influenza A is adapted to birds, it can infect people and sustain person-to-person transmission. It is a single-stranded RNA virus belonging to the orthomyxoviridae family [33]. The most well-known strain is H5N1, which was first isolated from a farmed goose in China in 1996 and caused the first human outbreak in Hong Kong in 1997. Since then, there have been reports of the disease in over 60 countries, primarily in Asia, Africa, the Pacific, Europe, and the Middle East [34]. Unlike COVID-19 and MERS, avian influenza more commonly affects the pediatric population, with almost half of the reported cases being <18 years old [35]. The clinical manifestations of H5N1 infection range from asymptomatic infection to severe pneumonia with multiorgan failure. Fatality rate of hospitalized patients is high, up to 50% [36]. According to the World Health Organization, H5N1 influenza should be considered in all patients presenting with fever, cough, sore throat, shortness of breath, and a positive contact history within 7 days of onset of symptoms with (1) a confirmed human case of H5N1 influenza during the infectious stage; (2) birds that are sick or have died of an unknown illness; (3) a recent visit to an area where there has been an outbreak of H5N1 avian influenza, or to an area where it is now considered endemic; and (4) individuals who have worked in a laboratory that is processing either suspected human or avian samples of the H5N1 infection [37].

The radiographic features of H5N1 pneumonia are reticular opacities with areas of patchy consolidations which rapidly become confluent on follow-up chest radiographs. H5N1 pneumonia is typically multifocal and bilateral, with a predilection for the lower lobes. During the course of the disease, bilateral pleural effusion and cavitations can appear [36–39]. CT is rarely performed during the acute stage of the disease. On CT, H5N1 pneumonia typically presents as multifocal or diffuse ground-glass opacities with areas of consolidations. CT findings during the convalescent stage can show signs of post-infective fibrosis [37, 38].

### Bacterial infections

#### *Streptococcus anginosus* group

*Streptococcus anginosus* group, previously known as *Streptococcus milleri*, is a group of three species of streptococci (*S. anginosus*, *S. constellatus*, and *S. intermedius*) that are commensals in the oral cavity and may cause opportunistic severe infections in the abdominal cavity, central nervous system, lung, and pleural space [40]. These pathogens have a striking predilection for abscess formation or empyema that usually require prolonged antibiotic therapy and surgical interventions [41]. While the *S. anginosus* group is increasingly recognized as a substantial cause of pulmonary infections in adults, especially immunocompromised patients, it is a rare cause of pulmonary infections in both immunocompromised and even immunocompetent children [42, 43].

**Fig. 3:**
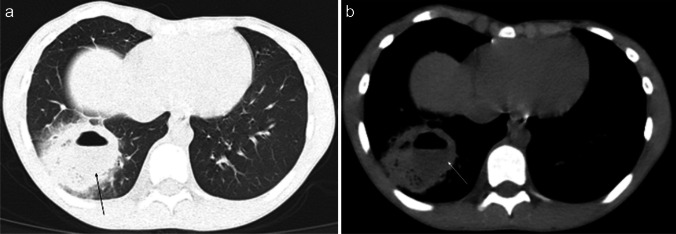
Axial non-contrast-enhanced computed tomography images in a 14-year-old boy with *Streptococcus anginosus* infection who presented with fever, cough, and right-sided chest pain. **a** Lung window image shows a round lesion with a thick wall, surrounding ground-glass opacities and an internal air-fluid level consistent with a lung abscess (*arrow*). **b** Mediastinal window image better demonstrates the air-fluid level within the lung abscess (*arrow*)

Affected patients with complicated pulmonary infection present with prolonged fever, respiratory symptoms, chest pain, and chest distress [40]. The most common findings on CT include lung abscesses, found in up to one-third of cases, and consolidations complicated by lung necrosis (Fig. 3). Pleural effusions are common, seen in up to two-thirds of cases. Pleural fluid is usually complex (loculated pleural fluid, with or without septations or split pleura sign), indicative of empyema. Hilar lymphadenopathy is occasionally seen [43]. Although rare, infection with the *S. anginosus* group should be considered in the differential diagnosis of pediatric patients presenting with empyema or lung abscess, regardless of their immune status.

#### Tularemia

Tularemia, also known as rabbit fever, is an infectious disease caused by the facultative intracellular gram-negative coccobacillus *Francisella tularensis*. It is a highly virulent zoonotic disease with sources of human infection in both wild and domestic animals, such as small rodents, rabbits, ticks, and mosquitoes, and in bodies of water. Infection may also occur through the skin, ingestion of undercooked meat, or inhalation of infected aerosols. It does not spread directly between people. *Francisella tularensis* can be divided into four subspecies: ssp. *tularensis*, *holarctica*, *mediasiatica*, and *novicida*. Highly virulent subspecies *tularensis* is found mainly in the USA and Canada, while the less virulent strains are present throughout the Northern Hemisphere [44].

Clinical disease in humans varies according to the mode of acquisition and is described as ulceroglandular/glandular, oropharyngeal, oculoglandular, typhoidal, or pneumonic tularemia. In children, the ulceroglandular/glandular form is the most common by far [45]. Primary pneumonic tularemia is rare. Tularemia pneumonia more commonly develops secondarily through hematogenous dissemination of ulceroglandular or typhoidal disease. Tularemia pneumonia typically presents with fever, general malaise, and respiratory symptoms [46, 47].

**Fig. 4:**
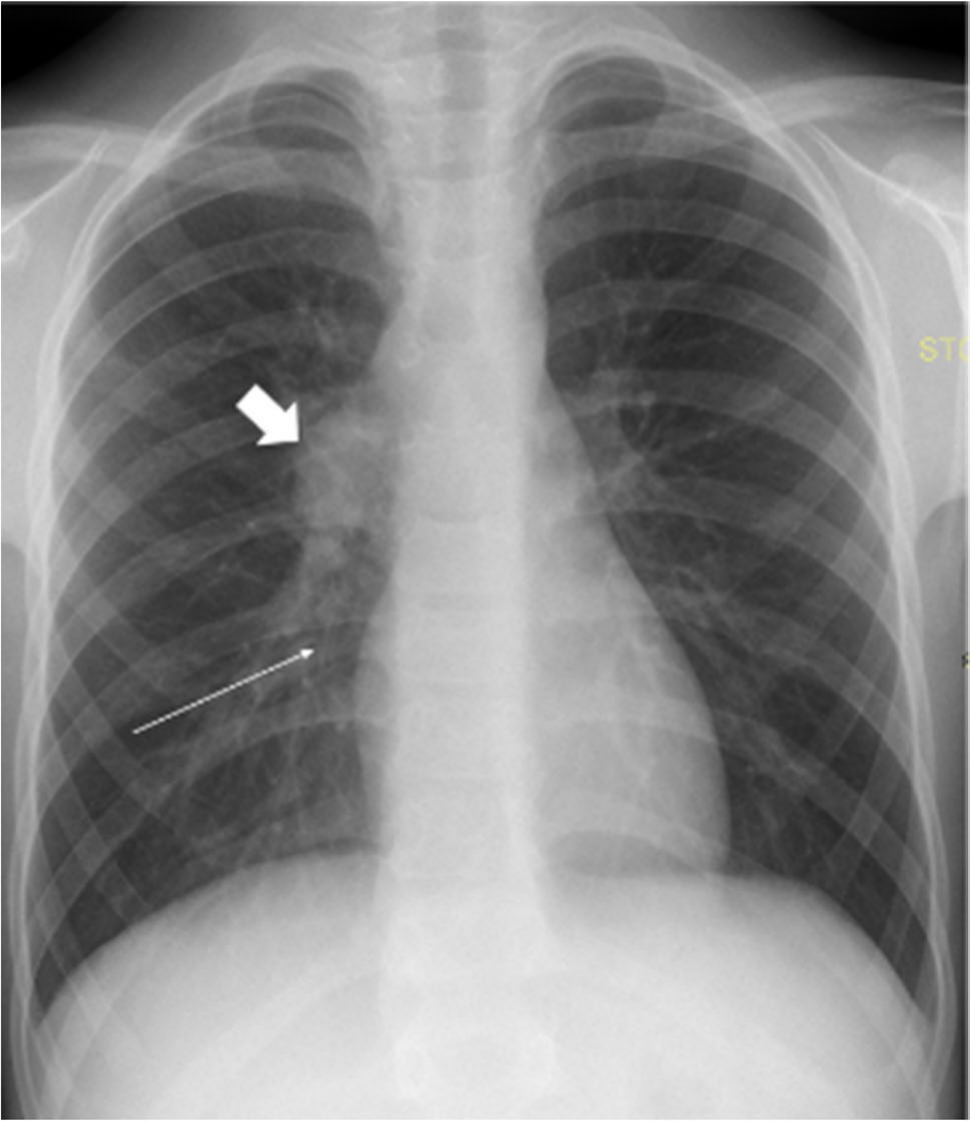
A posteroanterior chest radiograph in a 10-year-old boy with fever, cough, and malaise shows right hilar lymphadenopathy (*thick arrow*) and slight opacification within the right lower lung lobe (*thin arrow*). Ultrasonography (not shown) showed a small uncomplicated pleural effusion. Microbiological investigations confirmed tularemia

Tularemia pneumonia characteristically shows unilateral or bilateral lung consolidative opacities, hilar lymphadenopathy, and pleural effusion on chest radiography (Fig. 4). Chest CT often demonstrates dense lobar lung consolidation, usually in the periphery, and lymphadenopathy [48]. The complications of tularemia pneumonia include lung abscess and acute respiratory distress syndrome (ARDS) [48].

#### Psittacosis

Psittacosis, also known as ornithosis or parrot fever, is an infectious zoonotic disease caused by the obligate intracellular gram-negative bacterium *Chlamydia psittaci*. Birds are the most common reservoirs of the bacteria and direct contact with infected birds, exposure to their feces, or inhalation of contaminated aerosols may lead to human infection. Person-to-person transmission is rare [49]. Clinical presentation of *C. psittaci* infection can vary from subclinical or influenza-like symptoms to severe pneumonia complicated with multiple organ dysfunction syndrome. Fever, nonproductive cough, dyspnea, fatigue, headache, and myalgia are the most common symptoms of infection [50, 51]. Psittacosis typically affects bird owners, veterinarians, breeders and sellers of birds, and commercial poultry processors; children are rarely affected [52, 53].

**Fig. 5:**
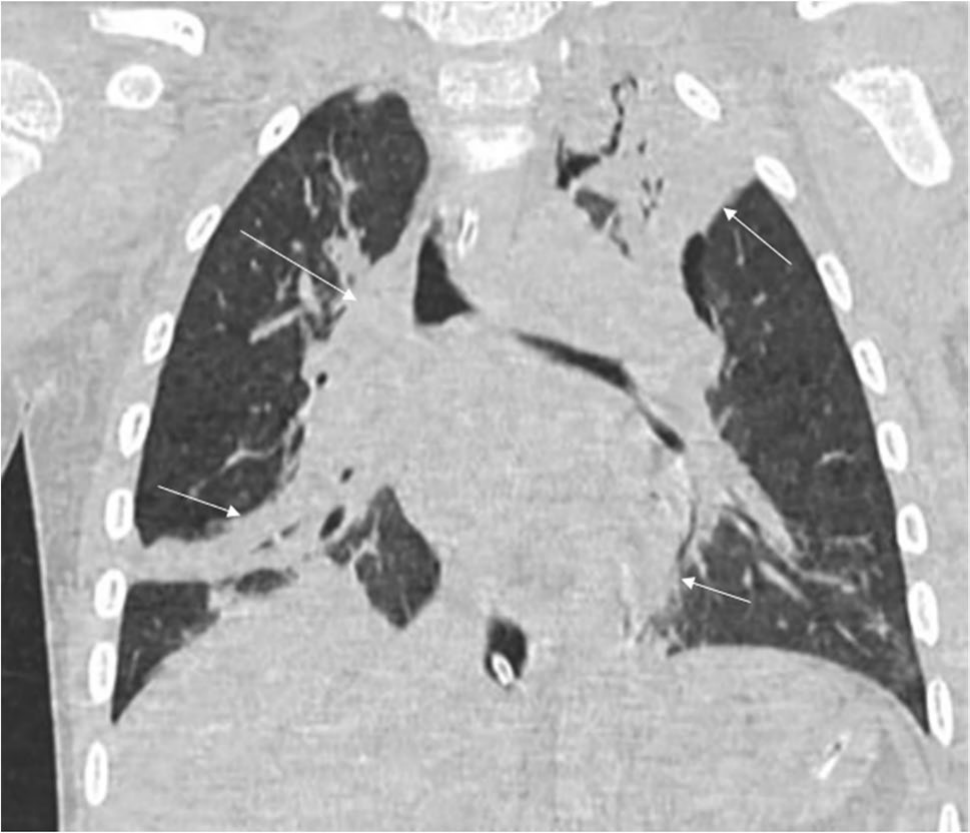
Coronal non-contrast-enhanced lung window computed tomography image in a 3-year-old boy affected by psittacosis who presented with severe dyspnea. The image shows multiple bilateral lung consolidations (*arrows*) (figure from “A pediatric case of *Chlamydia psittaci* caused severe Acute Respiratory Distress Syndrome (ARDS) in Italy” by Marchese et al.; licensed under CC BY; original annotated)

The radiological features of pneumonia caused by *C. psittaci* are large areas of consolidation or ground-glass opacities, typically bilateral and involving multiple lobes (Fig. 5). Mild to moderate pleural effusion is a common accompanying finding, while mild pericardial effusion is less commonly seen [49]. Neither the radiological features nor the clinical course is specific for diagnosis. Therefore, the potential of a history of contact with birds should be considered in any case of unexplained atypical pneumonia.

### Parasitic infections

#### Echinococcosis

Echinococcosis, also known as “hydatid disease,” is a parasitic infection caused by the tapeworm genus *Echinococcus* [54]. The disease occurs in most areas of the world; however, it is most prevalent in some areas of South America, Africa, and Eurasia [55]. Carnivores (mostly dogs) are the parasite’s definitive hosts and harbor the mature tapeworm in their intestines, while intermediate hosts are usually herbivores such as sheep or cattle. Humans act as accidental hosts who are infected through ingestion of the parasite’s eggs which have contaminated food, water, and soil [54]. Once ingested, the parasite penetrates the intestine to enter the portal system. The liver acts as the first line of defense, and is therefore the organ most commonly affected by this disease (75%); the second most commonly affected organ is the lung (15%).

The disease develops as a slow-growing cystic mass in the affected organ. The initial phase of primary infection is asymptomatic. Many infections are acquired in childhood but do not cause clinical manifestations until adulthood. Pulmonary disease clinically manifests earlier than the disease in the liver; therefore, it is more commonly observed in children [56]. Pulmonary echinococcosis typically presents with chest pain, dry cough, shortness of breath, and hemoptysis, due to the mass effect caused by the cyst. Acute-onset of more severe symptoms, including anaphylactic reaction, may suggest cyst rupture, in some cases patients can expectorate “grape skin”-like material [57, 58].

**Fig. 6:**
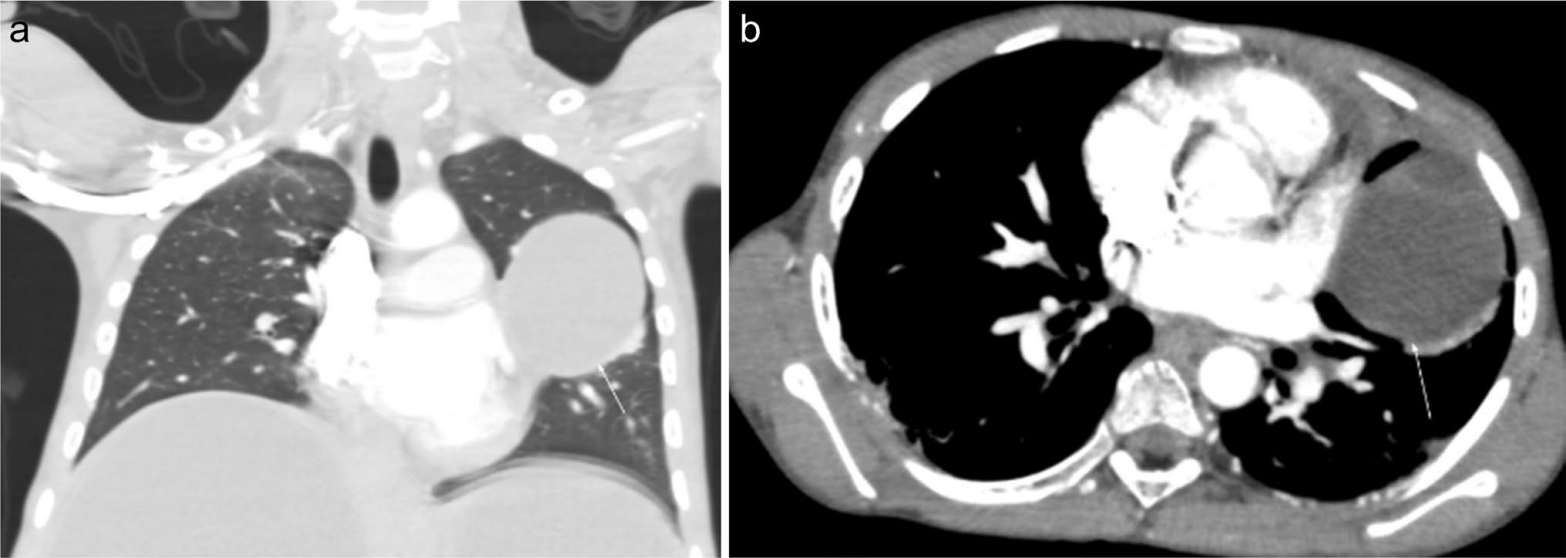
Contrast-enhanced computed tomography images in an 11-year-old boy with hydatid disease who presented to hospital with a 2-month history of hemoptysis, abdominal distention, and elevated inflammatory markers. The patient had a history of travel to an endemic area in the 5 months prior to presentation. Coronal lung window (**a**) and axial mediastinal window (**b**) images demonstrate a well-defined cystic mass (*arrows*) in the mid zone of the left lung. The cyst wall has no calcification and enhances after contrast agent injection, while the content of the cyst remains homogenously hypodense

On chest radiography, an uncomplicated pulmonary hydatid cyst appears as a well-defined homogenous opacity, more commonly appearing in the lower lobes (Fig. 6). There can be multiple cysts in 30% of cases and appear bilaterally in 20% of cases. Calcification in the cyst wall makes the diagnosis almost certain; however, it is rarely seen. Complicated cysts (i.e., eroding a bronchus) can have a more heterogeneous appearance with deformed shape, air inclusions, and air-fluid levels. In such cases, CT can be especially helpful in establishing the diagnosis [57].

#### Paragonimiasis

Paragonimiasis, also known as lung fluke disease, is a parasitic disease caused by several species of lung flukes belonging to genus *Paragonimus*, most predominantly *P. westermani*. The parasite is endemic to Southeast Asia, the Indian subcontinent, South and North America, and Africa [59]. Infection is acquired through consumption of improperly cooked crab, crayfish, or raw meat of wild boar or deer infected with immature forms of lung flukes called metacercariae; tailless, encysted late larvae [60]. After ingestion, the immature forms migrate through the duodenal wall, peritoneal cavity, and diaphragm to become encapsulated and mature within the host, typically in the lungs, or less commonly other organs, including the liver, brain, kidneys, adrenal glands, and lymph nodes.

Common symptoms of paragonimiasis include fever, fatigue, cough, diarrhea, and abdominal pain, while neurological symptoms are far less common. Subcutaneous nodules and hepatomegaly can be recognized during physical examination. Diagnosis is often difficult to establish because of the vague and non-specific symptoms, especially in children who cannot accurately describe their symptoms and dietary history [60].

**Fig. 7:**
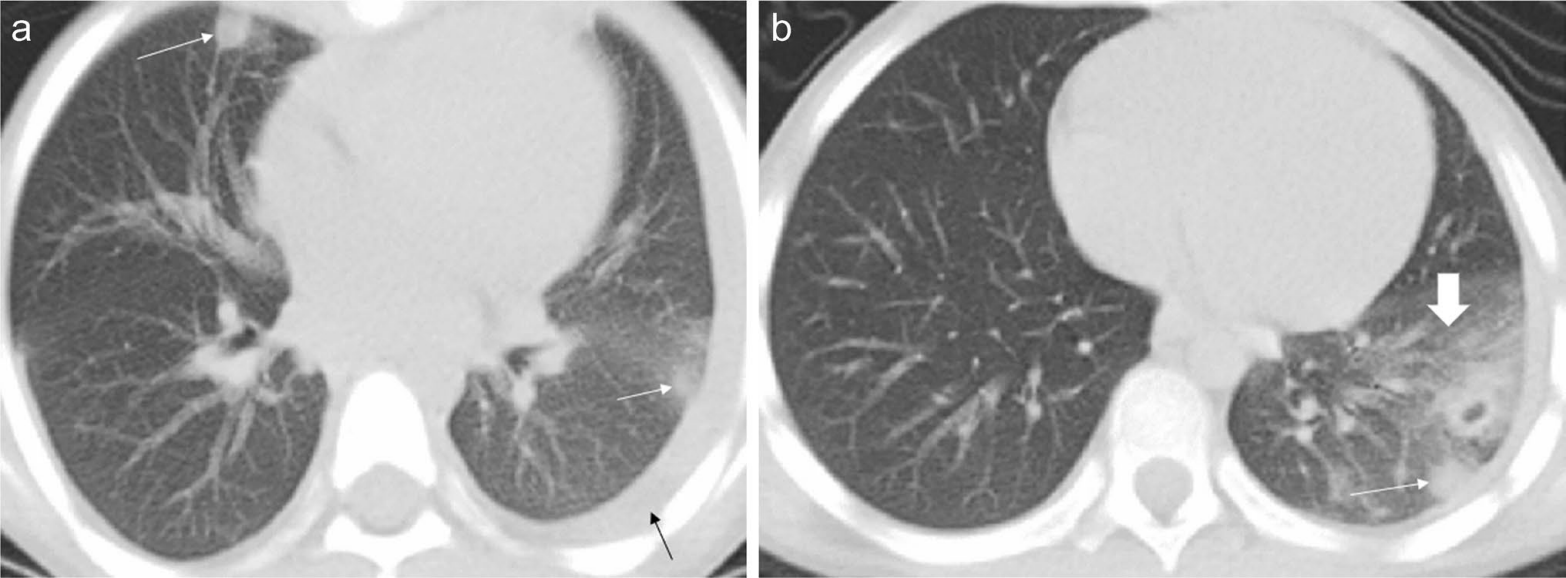
Axial non-contrast-enhanced lung window computed tomography images in a 7-year-old girl with paragonimiasis who presented to hospital with abdominal pain and subcutaneous nodules. **a** Image at the level of the mid lung zone shows ill-defined lung nodules (*white arrows*) and a left-sided pleural effusion (*black arrow*). **b** Image at the level of the lower lung zone shows ill-defined lung nodules (*thin arrow*) and alveolar opacification (*thick arrow*) (figure from “A retrospective clinical analysis of pediatric paragonimiasis in a Chinese children’s hospital from 2011 to 2019” by Qian et al.; licensed under CC BY 4.0; original cropped and annotated)

Chest radiography and CT can be helpful in reaching the diagnosis. Typical chest radiographic findings are pleural effusion, hilar lymphadenopathy, and patchy pulmonary consolidations with ground-glass opacities. On CT, ill-defined pulmonary nodules and consolidation with round low-attenuation cystic lesions filled with fluid or gas can be seen (Fig. 7); intra-cystic worms may be detected [60–62]. When children from endemic areas present with respiratory symptoms, laboratory findings of eosinophilia, and imaging demonstrations pleural effusion and lung consolidations with internal cystic lesions, diagnosis of paragonimiasis should be strongly considered.

#### Amoebiasis

Amoebiasis is caused by the parasitic amoeba *Entamoeba histolytica*. Amoebiasis is a common parasitic disease in tropical and developing areas, such as Central and South America, Africa, and the Indian Subcontinent, due to lower levels of sanitation, hygiene, and socio-economic status. More recently, with wide-spread global tourism, amoebiasis is becoming a globalized disease [63, 64]. *Entamoeba histolytica* has two life cycles, one as an infectious trophozoite and the second as a cyst that can survive for months in harsh environments outside the host’s body. The most common route of infection is by ingestion of a mature cyst through contaminated food, water, hands, or sexual contact. Trophozoites release from the cyst in the small intestine and multiply in the lumen of the intestine. Some inhabit the intestinal lumen causing noninvasive infection that enables further spread of the disease, while others invade the intestinal mucosa and cause gastrointestinal disease, or further enter blood vessels and infect extraintestinal organs such as the liver, brain, and lungs [64].

Extraintestinal amoebiasis varies from the most frequent amoebic liver abscess to pulmonary amoebiasis, cardiac infection, or even brain infection. Most extraintestinal amoebiasis begins in the form of an amoebic liver abscess and spreads directly to adjacent organs. Hematogenous spread without hepatic involvement is also possible [65]. Typical symptoms of pulmonary amoebiasis include fever, productive cough, fatigue, and dyspnea. The characteristic imaging feature of pulmonary amoebiasis is abscess formation. When a lung abscess (amoeboma) is formed and connects to the bronchial tree, there is the production of characteristic brown-colored sputum, often clinically referred to as “anchovy sauce sputum” [64].

**Fig. 8:**
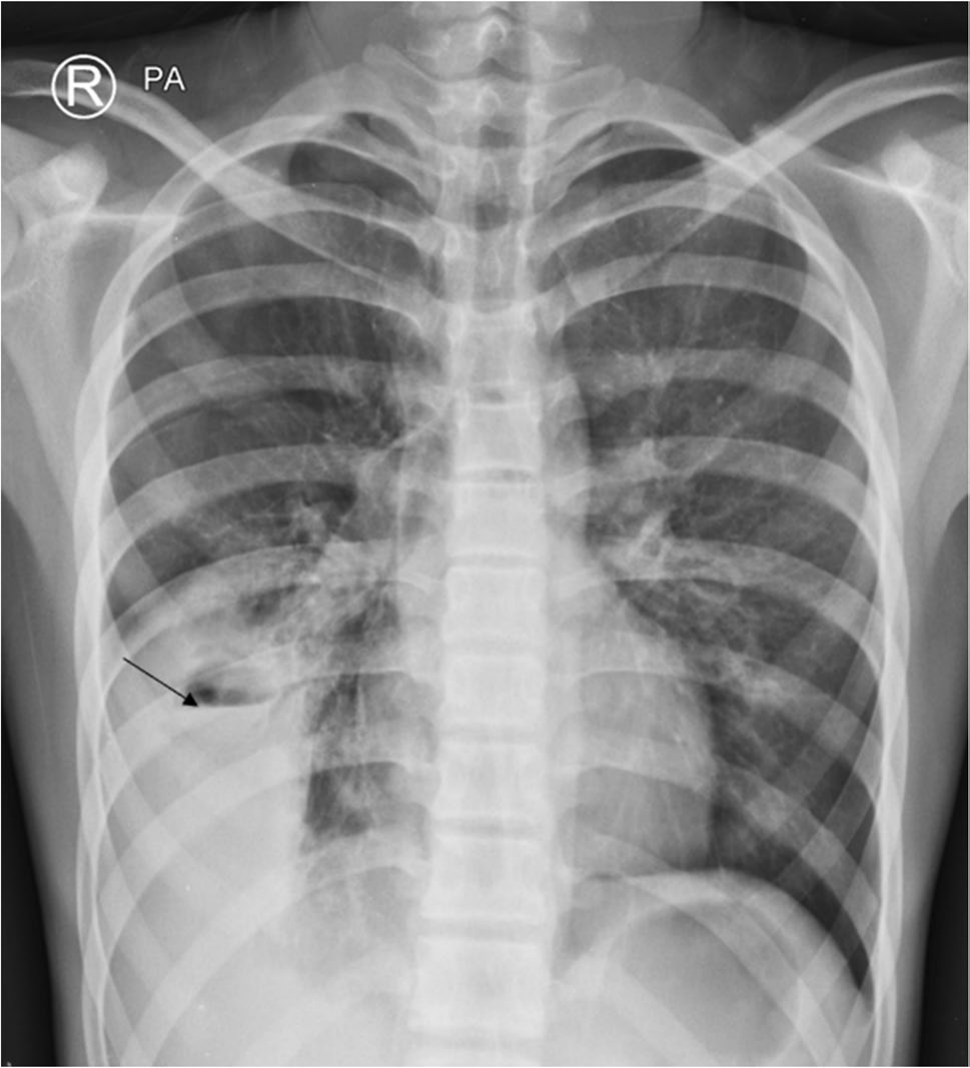
Posteroanterior chest radiograph in a 15-year-old boy with amoebiasis who presented to the emergency department complaining of breathlessness for the preceding 10 days shows opacity with an air-fluid level (*arrow*) in the right lower lobe (amoeboma) and a right-sided pleural effusion (figure from “A Rare Case of Primary Pulmonary Amoebiasis without Gastrointestinal Involvement: A Case Report” by Nugroho et al.; licensed under CC BY-SA; original cropped and annotated)

Chest radiography is the first-line radiologic imaging that can raise suspicion of pulmonary amoebiasis when lung abscess is found. The amoebic abscess is characteristically amorphous in shape, with thick irregular walls, with or without an air-fluid level (Fig. 8). Before the formation of the amoebic abscess, consolidation within the lung and pleural effusion is seen [62]. In a country where amoeba is endemic, the differential diagnosis for thoracic abscess should include amoebic origin, especially in the presence of characteristic imaging features [66].

### Mimics of unusual lung infections in children

#### Hypersensitivity pneumonitis

Hypersensitivity pneumonitis, also previously known as extrinsic allergic alveolitis, is a complicated and heterogeneous interstitial lung disease caused by an excessive immune response to an inhaled antigen in susceptible individuals appearing as inflammation and/or fibrosis of the lung parenchyma and small airways. Hypersensitivity pneumonitis has a predilection for women and older individuals; however, it is the most frequent chronic interstitial lung disease in children [67]. Although more than 200 sensitizing antigens have been identified, bird antigens are by far the most common causative antigens in children [67]. Accurate diagnosis of hypersensitivity pneumonitis is difficult and necessitates a detailed exposure history, as well as a multidisciplinary clinical, histopathologic, and radiologic evaluation.

The diversity of clinical manifestations depends on the nature of the causal agent, the duration of exposure, and host factors. The disease can be classified as acute/inflammatory or chronic/fibrotic form. The acute/inflammatory form is denoted by a disease lasting <6 months and is often reversible. In this form, typical symptoms are dyspnea, cough, and, less commonly, wheezing and weight loss. Chronic/fibrotic form is characterized by a disease lasting >6 months and the presence of fibrotic changes on CT imaging or histology. In this form, typical symptoms are gradual respiratory failure with chronic dry cough and weight loss [68].

**Fig. 9:**
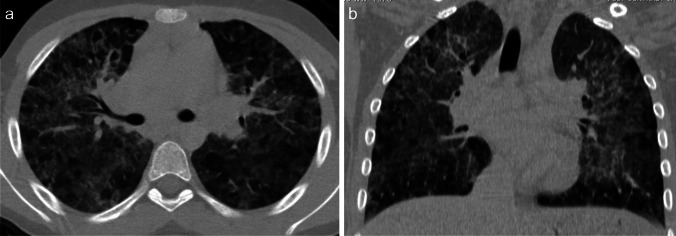
Axial (**a**) and coronal (**b**) contrast-enhanced lung window computed tomography images in a 9-year-old girl with a 3-month history of chronic dry cough, wheezing, and loss of weight demonstrate patchy bilateral ground-glass opacities, small centrilobular nodules, lobular areas of reduced attenuation and vascularization (i.e., mosaic attenuation), and interlobular septal thickening with sparing of the lung bases. No associated features of chronic lung disease. Lung biopsy confirmed hypersensitivity pneumonitis. On further investigation, her mother also had similar clinical symptoms. The family had started breeding pigeons 6 months prior. When the antigen was removed, the child’s symptoms completely resolved, and follow-up chest radiography performed 3 months later (not shown) was normal

Radiological findings of hypersensitivity pneumonitis vary according to the stage of the disease. Although chest radiography can help identify the disease, CT is increasingly used, as it is more sensitive for subtle ground-glass opacities and identification of fibrosis [69]. In the acute/inflammatory form, CT typically shows patchy or diffuse bilateral ground-glass opacities, small centrilobular nodules, lobular areas of reduced attenuation and vascularization (i.e., mosaic attenuation), and air trapping on expiratory images (Fig. 9). Thickening of the interlobular septa, traction bronchiectasis, and a subpleural honeycomb pattern are signs of chronic/fibrotic form. The lung bases are typically spared [70].

#### Pulmonary hemorrhage

Pulmonary hemorrhage is extravasation of blood into airways and/or lung parenchyma. It is an uncommon, potentially life-threatening event that affects children of all ages and can be associated with a wide range of diseases. It characteristically presents with hemoptysis, although some children have a more elusive presentation with cough, tachypnea, dyspnea, wheezing, and even fever [71]. Although pulmonary hemorrhage can be localized or diffuse, the localized form is more common. There are numerous causes for localized pulmonary hemorrhage, such as infection, congenital malformation, vascular anomalies, bleeding diathesis, trauma, and foreign body. Diffuse pulmonary hemorrhage is usually associated with an underlying systemic disease (e.g., vasculitis, idiopathic pulmonary hemosiderosis, coagulopathy, celiac disease, post-bone marrow transplantation, drugs, pulmonary veno-occlusive disease, pulmonary hypertension) [72].

**Fig. 10:**
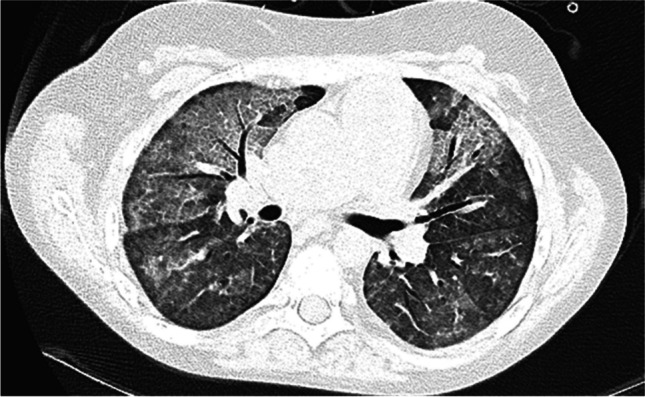
Axial contrast-enhanced lung window computed tomography image in a 12-year-old girl with granulomatosis, polyangiitis, and pulmonary hemorrhage who presented with hemoptysis and anemia. The image shows interlobular septal thickening superimposed on areas of ground-glass opacity, resulting in a characteristic crazy-paving pattern

There are no pathognomonic radiographic findings for pulmonary hemorrhage. On radiographs, localized hemorrhage may appear as patchy alveolar opacities or dense consolidation. Diffuse hemorrhage appears as a symmetric diffuse hazy ground-glass pattern, which typically predominates in the lower lung zones with sparing of the apices and costophrenic angles. Diffuse pulmonary hemorrhage secondary to vasculitis may have an asymmetric or patchy distribution [73]. A characteristic feature of hemorrhage is rapid resolution of radiological opacities over 2–3 days, unless hemorrhage is ongoing or repetitive [71]. As hemorrhage clears, an interstitial pattern with thickening of the interlobular septa may emerge. Within a couple of weeks, chest radiographs typically normalize. However, following repeated episodes of pulmonary hemorrhage, a reticulonodular pattern of interstitial fibrosis develops. The CT findings of pulmonary hemorrhage correlate well with chest radiography. CT is a useful adjunct to radiography in the evaluation of localized pulmonary hemorrhage for depiction of the underlying disease and is also a valuable imaging modality for embolization planning in patients with massive hemorrhage (Fig. 10). Findings suggestive of pulmonary vasculitis on CT include fluffy, centrilobular, perivascular densities found centrally and peripherally. Patients with a normal chest radiograph and a high suspicion of pulmonary hemorrhage may benefit from CT for the diagnosis and differentiating it from other potential diagnostic considerations [71].

#### Idiopathic eosinophilic pneumonia

Eosinophilic pneumonias are a large group of interstitial lung diseases characterized by a substantial infiltration of the alveolar spaces and lung interstitium by eosinophils, with conservation of the lung structure. Idiopathic eosinophilic pneumonias are rare entities and pediatric case descriptions are scarce. Diagnosis of eosinophilic pneumonia relies on the demonstration of alveolar eosinophilia on bronchoalveolar lavage and elimination of a secondary cause (parasitic or fungal infection, asthma, cystic fibrosis, drug effect). Idiopathic eosinophilic pneumonias can have acute and chronic presentations. Acute eosinophilic pneumonia clinically presents as a hypoxic and febrile acute respiratory distress syndrome, accompanied by myalgia. Chronic eosinophilic pneumonia has a slower progressive clinical presentation beginning with prolonged dry cough [74].

**Fig. 11:**
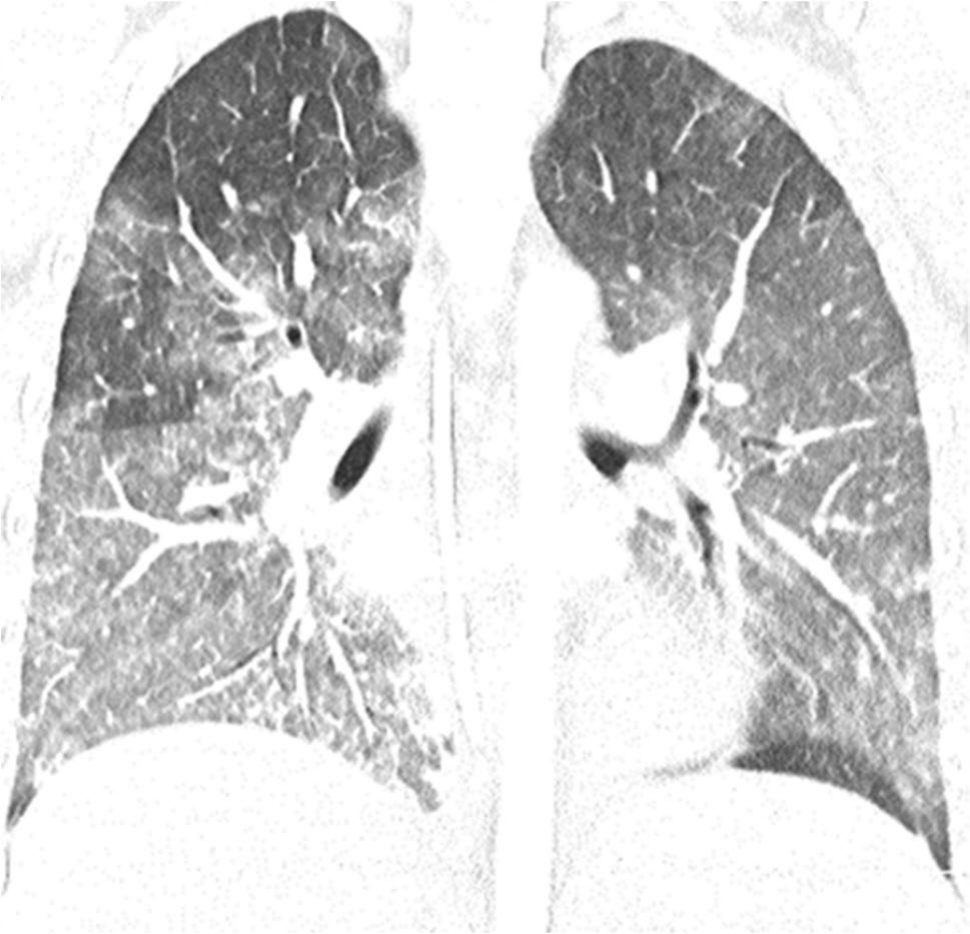
Coronal contrast-enhanced lung window computed tomography image in a 15-year-old boy with acute eosinophilic pneumonia who presented with progressively worsening respiratory symptoms and fever for 3 days, hypoxemia, bronchoalveolar lavage fluid consisting of >25% eosinophils, and lack of underlying infections or other potential lung disorders. The image shows diffuse air-space opacities in both lungs in keeping with eosinophilic pneumonia. The patient responded well to corticosteroid treatment

The radiographic pattern of acute eosinophilic pneumonia is nonspecific. Diffuse interstitial and air-space opacities without peripheral predominance, interlobular septal thickening, and pleural effusions can be observed. The random distribution of abnormalities and pleural effusion are important diagnostic features [75]. The radiographic pattern in the chronic form is more specific with non-segmental peripheral airspace consolidation (i.e., reverse batwing appearance) involving mainly the middle and upper lobes. In the later stages of eosinophilic pneumonia, ground-glass opacities, pulmonary nodules, and reticulation can be demonstrated on chest CT (Fig. 11). Pleural effusion is not a common finding [76].

## Conclusion

The clinical presentation of children with new and unusual lung infections is often non-specific. Therefore, imaging evaluation plays an important role in initial detection, follow-up for disease progression, and assessment of potential complications of new and unusual pediatric lung infections. Clear and up-to-date understanding of the imaging characteristics of unusual pediatric lung infections and potential mimics is essential for radiologists to provide an accurate and clinically useful differential diagnosis. This, in turn, can lead to timely diagnosis and optimal management of pediatric patients with unusual lung infections.
